# Structural Basis for Carbapenem-Hydrolyzing Mechanisms of Carbapenemases Conferring Antibiotic Resistance

**DOI:** 10.3390/ijms16059654

**Published:** 2015-04-29

**Authors:** Jeong Ho Jeon, Jung Hun Lee, Jae Jin Lee, Kwang Seung Park, Asad Mustafa Karim, Chang-Ro Lee, Byeong Chul Jeong, Sang Hee Lee

**Affiliations:** National Leading Research Laboratory of Drug Resistance Proteomics, Department of Biological Sciences, Myongji University, 116 Myongjiro, Yongin, Gyeonggido 449-728, Korea; E-Mails: najashil@hanmail.net (J.H.J.); topmanlv@hanmail.net (J.H.L.); leo102@naver.com (J.J.L.); ryduses@naver.com (K.S.P.); asadmustafa8@gmail.com (A.M.K.); crlee@mju.ac.kr (C.-R.L.); bcjeong@mju.ac.kr (B.C.J.)

**Keywords:** carbapenemases, carbapenems, structure, catalytic mechanism

## Abstract

Carbapenems (imipenem, meropenem, biapenem, ertapenem, and doripenem) are β-lactam antimicrobial agents. Because carbapenems have the broadest spectra among all β-lactams and are primarily used to treat infections by multi-resistant Gram-negative bacteria, the emergence and spread of carbapenemases became a major public health concern. Carbapenemases are the most versatile family of β-lactamases that are able to hydrolyze carbapenems and many other β-lactams. According to the dependency of divalent cations for enzyme activation, carbapenemases can be divided into metallo-carbapenemases (zinc-dependent class B) and non-metallo-carbapenemases (zinc-independent classes A, C, and D). Many studies have provided various carbapenemase structures. Here we present a comprehensive and systematic review of three-dimensional structures of carbapenemase-carbapenem complexes as well as those of carbapenemases. We update recent studies in understanding the enzymatic mechanism of each class of carbapenemase, and summarize structural insights about regions and residues that are important in acquiring the carbapenemase activity.

## 1. Introduction

β-Lactamases are bacterial enzymes that hydrolytically inactivate β-lactam antibiotics and are a major cause of the emergence of pathogenic bacteria resistant to β-lactam antibiotics such as penicillins, cephalosporins, monobactams, and carbapenems. Based on the sequence homology, β-lactamases are grouped into four molecular classes A, B, C, and D [1]. Classes A, C, and D of β-lactamases are serine-based enzymes in which a covalent acyl-enzyme intermediate is formed. A conserved serine in the active site acts as the nucleophile to attack the β-lactam C–N bond. The acyl-enzyme intermediate formed by the acylation reaction is hydrolyzed by a conserved deacylating water molecule, and then the hydrolyzed product is released from the active site. However, class B β-lactamase is a metallo-β-lactamase that relies on a water molecule coordinated to a divalent cation (Zn^2+^) to activate and break the β-lactam ring. In these enzymes, the covalent acyl-enzyme intermediate is not formed.

**Figure 1:**
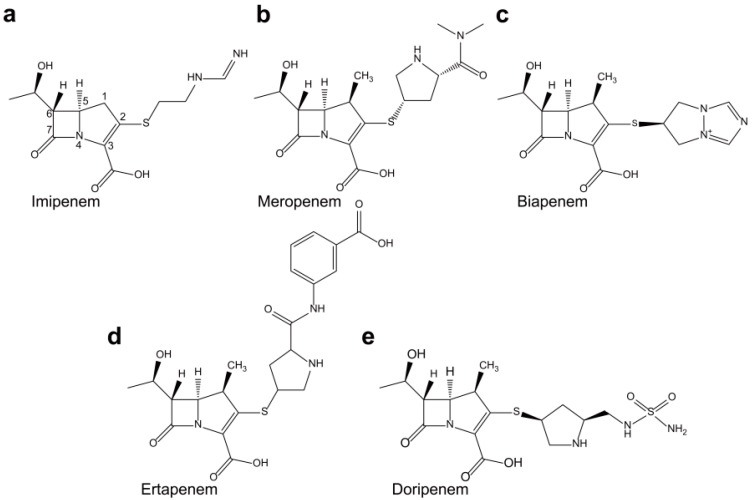
Chemical structures of (**a**) imipenem; (**b**) meropenem; (**c**) biapenem; (**d**) ertapenem; and (**e**) doripenem. The β-lactam nucleus is numbered.

Carbapenemases are the most versatile family of β-lactamases and are able to hydrolyze carbapenems and other β-lactams [2]. Carbapenems (imipenem, meropenem, biapenem, ertapenem, and doripenem) have a penicillin-like five-membered ring, but the sulfur at C-1 in the five-membered ring is replaced with a carbon atom and a double bond between C-2 and C-3 is introduced (Figure 1) [3]. They have the broadest spectra of antimicrobial activity among all β-lactams and are primarily used to treat infections by aerobic Gram-negative bacteria. The emergence and spread of acquired carbapenem resistance due to carbapenemases are a major concern of the public health and is considered a global sentinel event [4]. According to their dependency on divalent cations for enzyme activation, carbapenemases can be divided into non-metallo-carbapenemases (zinc-independent classes A, C, and D) and metallo-carbapenemases (zinc-dependent class B) [5].

Class A carbapenemases, including the KPC (*Klebsiella pneumoniae* carbapenemase), IMI (imipenem-hydrolyzing β-lactamase), SME (*Serratia marcescens* enzyme), SFC (*Serratia fonticola* carbapenemase), NMC-A (non-metallo carbapenemase of class A) families, and some GES (Guiana extended-spectrum β-lactamase) enzymes, have been most frequently discovered in isolates from *Enterobacteriaceae* and in species such as *Pseudomonas* *aeruginosa* [5,6,7]. These enzymes are inhibited by clavulanate, except for some KPC type enzymes such as KPC-2, and hydrolyze penicillins or cephalosporins more efficiently than carbapenems. Class B carbapenemases, including VIM (Verona integron-encoded metallo-β-lactamase), IMP (imipenem-resistant *Pseudomonas*), and SPM-1 (Sao Paulo metallo-β-lactamase) families, have been previously detected in strains of *P*. *aeruginosa*, *Acinetobacter baumannii*, and members of the *Enterobacteriaceae* family [6]. These carbapenemases hydrolyze penicillins, cephalosporins, and carbapenems; however, they lack the ability to hydrolyze aztreonam. Class C carbapenemases, such as ACT-1 (AmpC-type β-lactamase), DHA-1 (Dhahran Hospital in Saudi Arabia β-lactamase), CMY-2 (cephamycin-hydrolyzing β-lactamase), and CMY-10, were identified in *Enterobacteriaceae* [8,9,10,11]. They are plasmid-encoded class C β-lactamases that exhibit the catalytic activity for imipenem [8]. Recently, ADC-68 was identified from carbapenem-resistant *A. baumannii* D015 [12]. Class D carbapenemases belong to the OXA (oxacillinase) family and were identified in *Acinetobacter* clinical isolates [13]; they hydrolyze carbapenems weakly and are poorly inhibited by clavulanate [6].

In this review, to understand the enzymatic mechanism of the four classes of carbapenemases, three-dimensional structures of carbapenemases (in brief) and carbapenemase-carbapenem complexes (in detail) are discussed, with special attention to studies published from 2000 and 2014.

## 2. Non-Metallo-Carbapenemases: Zinc-Independent Classes A, C, and D

### 2.1. Class A Carbapenemases

A phylogenetic analysis of class A carbapenemases together with 62 representative class A β-lactamases showed that carbapenemases form six distantly related branches: IMI/NMC-A enzymes, SME enzymes, GES enzymes, KPC enzymes, SFC-1, and SHV-38 [14]. The clusters share amino acid sequence identities ranging from 32% to 70%. The first chromosome-encoded class A carbapenemase (NMC-A: non-metallo carbapenemase of class A) was identified from the *Enterobacter cloacae* strain isolated from the pus of a fistulized subcutaneous abscess of a patient hospitalized in Paris [15]. The IMI enzymes (imipenem-hydrolyzing β-lactamases) was identified from rare isolates of *Enterobacter* in the USA [16], France [17], Croatia [18], Finland [19], Argentina [20], and Ireland [21]. Most *bla*_IMI-1_ genes are located on chromosome and associated with *imi*-*R* gene coding for a LysR transcriptional regulator, which limits their spread and their expression at a high level. The plasmid-located *bla*_IMI-2_ gene was discovered in environmental *Enterobacter asburiae* strains from several US rivers [22] and in a single *E. cloacae* isolate in China [23]. The SME enzymes (*Serratia marcescens* enzymes), SME-1 to SME-5, have been found exclusively in *S. marcescens* and the five variants differ from each other by one to three amino acid substitutions. They are chromosomally encoded [24] and have been recovered sporadically throughout USA and Canada [24,25,26]. The GES (Guiana extended-spectrum β-lactamase) family enzymes include 26 variants [27] and differ from each other by one to four amino acid substitutions [28]. GES-1 of the GES family, not a carbapenemase, was identified from *Klebsiella pneumoniae* isolate in 2000 [29]. Some GES enzymes such as GES-2, GES-4, GES-5, GES-6, GES-11, GES-14, and GES-18 are able to hydrolyze imipenem [30]. GES-2 and GES-5 among them have a considerable carbapenemase activity. GES-2 was identified from *P. aeruginosa* [31] and GES-5 was identified from *Enterobacteriaceae* and *P. aeruginosa* [32,33]. KPC enzymes (*Klebsiella pneumoniae* carbapenemases) were mainly identified from *K. pneumoniae* and are currently the most clinically significant enzymes among the class A carbapenemases owing to conferring high levels of resistance to carbapenems as well as most β-lactams [34]. There are now 22 KPC variants and they differ from each other by one to three amino acid substitutions. The amino acids of KPC enzymes are similar to that of SFC-1 (*Serratia fonticola* carbapenemase) from *Serratia fonticola* (approximately 61% identity) [14]. SFC-1 was identified from *S. fonticola* strain isolated from an environmental isolate in Portugal [7]. SHV-38 was identified from *K. pneumoniae* and differs by a single substitution from the non-carbapenemase of class A, SHV-1 [35].

### 2.2. Structural Components and Catalytic Mechanism of Class A Carbapenemases

Class A β-lactamases possess four important structural motifs, such as Ser70-X-X-Lys73, Ser130-Asp131-Asn132, Lys234-Thr/Ser235-Gly236, and the Ω-loop. Ser70 acts as a nucleophile to attack the carbonyl carbon of the β-lactam ring, and the acyl-enzyme intermediate is subsequently formed. It was proposed that Lys73 and Glu166 act as the general base in the acylation or the deacylation step. Lys73, Lys234, and Ser130 are involved in the formation of a hydrogen bond network with a water molecule that is important in the deacylation step.

Crystal structures of class A carbapenemases, such as NMC-A, the SME-1, GES-5, SFC-1, and KPC-2, have been previously determined [36,37,38,39,40]. The catalytic efficiencies (*K*_cat_/*K*_m_) of NMC-A [41], SME-1 [42], GES-5 [43], SFC-1 [44], and KPC-2 [45] for imipenem were 11.3, 0.515, 0.286, 0.659, and 0.295 µM^−1^·S^−1^, respectively. Their overall structures were conserved in other typical class A β-lactamases and consisted of two main domains. Domain 1 consists of only α-helix and domain 2 comprises five β-strands containing α-helices. Their active-site motifs, Ser70-X-X-Lys73, Ser130-Asp131-Asn132, and Lys234-Thr/Ser235-Gly236 (Amber numbering scheme [46]), were conserved in all class A β-lactamases and carbapenemases. Interestingly, except for SHV-38, all class A carbapenemases contained only two cysteine residues (Cys69 and Cys238) which form a unique disulfide bridge [36,37,38,39,40]. The enzymatic mechanism of the class A carbapenemase for carbapenem is similar to that of the class A β-lactamase for other β-lactam antibiotics. It includes acylation and deacylation steps. In the acylation step, the active site Ser70 attacks the amide bond of the β-lactam substrate and forms an acyl-enzyme complex. To activate the Ser70 hydroxyl side chain, Glu166 and Lys73 share the role of the general base in either a competitive or a cooperative manner. In the deacylation step, the acyl adduct is hydrolyzed by a deacylating water molecule, and then the hydrolyzed product is released from the active site. Through this step, Ser70 is regenerated. The role of Glu166 (general base) is the activation of a deacylating water molecule to hydrolyze the acyl adduct [37,47,48,49,50,51,52].

**Figure 2:**
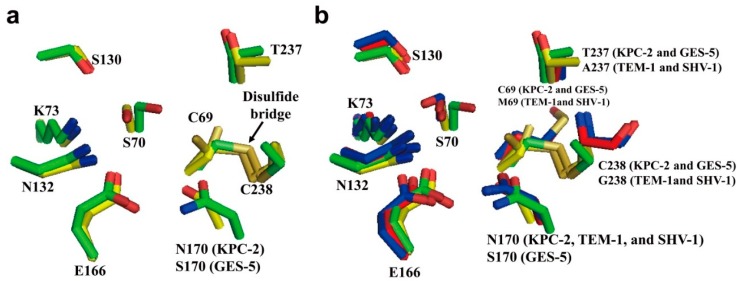
(**a**) Superposition of active sites of KPC-2 (PDB entry 2OV5, green) and GES-5 (PDB entry 4GNU, yellow) is shown. The disulfide bridge between C69 and C238 (arrow) is shown in KPC-2 and GES-5; (**b**) Superposition of active sites of class A carbapenemases (KPC-2 (PDB entry 2OV5, green) and GES-5 (PDB entry 4GNU, yellow)) and class A non-carbapenemases (TEM-1 (PDB entry 1ZG4, red) and SHV-1 (PDB entry 1SHV, blue)). The residues (C69/M69, S70, K73, S130, N132, E166, N170/S170, T237/A237, and C238/G238) in the active-site cleft are shown as sticks. Superpositions were performed using SSM Superpose [53] to align the complete chains. This figure was prepared using *PyMOL* [54].

The active site of class A carbapenemases is composed of several highly conserved residues, including Cys69, Ser70, Lys73, Ser130, Asn132, Glu166, Asn170, Thr237, and Cys238 (Amber numbering scheme [46]) (Figure 2a) [38]. In the case of GES-5, Asn170 is placed by Ser170 (Figure 2a) [36]. Many active site residues (Ser70, Lys73, Ser130, Asn132, and Glu166) are highly conserved among class A carbapenemases, but some residues are poorly conserved. For example, Cys69, Thr237, and Cys238 residues in KPC-2 and GES-5 are replaced by Met69, Ala237, and Gly238 in TEM-1 and SHV-1, respectively (Figure 2a,b) [36,38]. Therefore, KPC-2 and GES-5 contain the disulfide bond formed by C69 and C238, whereas this disulfide bond is not present in SHV-1 and TEM-1.

The comparison of the NMC-A structure with other class A β-lactamases structures revealed specific positional differences at an Asn132 residue of the conserved Ser130-Asp131-Asn132 motif and at 237–240 residues adjacent to the Lys234-Thr235-Gly236 motif. Swarén *et al.* [40] suggested that these differences provide a critical additional space, which might permit the access of the 6α-1*R*-hydroxyethyl group (the R1 side chain) of carbapenems.

In SME-1, the structural feature showed that the shorter distance between the Ser70 and Glu166 residues than other class A β-lactamases changes the position of the essential catalytic water molecule. In addition, Sougakoff *et al.* [39] suggested that the Ser237 in SME-1 has a significant effect on catalytic activity against imipenem because the side chain of Ser237 which is strongly stabilized by two hydrogen bonds with Arg220 contributes to the SME-1 oxyanion hole together with Ser70. Mutagenesis study also showed that the Ser237Ala mutation in SME-1 resulted in a specific five-fold decrease in catalytic activity against imipenem [55]. Moreover, to prove whether the disulfide bridge in SME-1 plays a crucial role in hydrolyzing imipenem, they made the Cys69Ala mutant of SME-1 by site-directed mutagenesis. The mutant protein was unable to confer resistance to imipenem [39].

**Figure 3:**
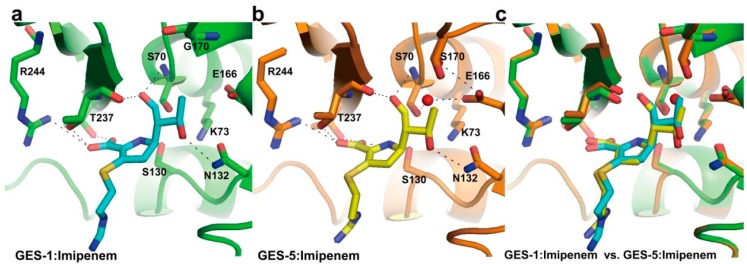

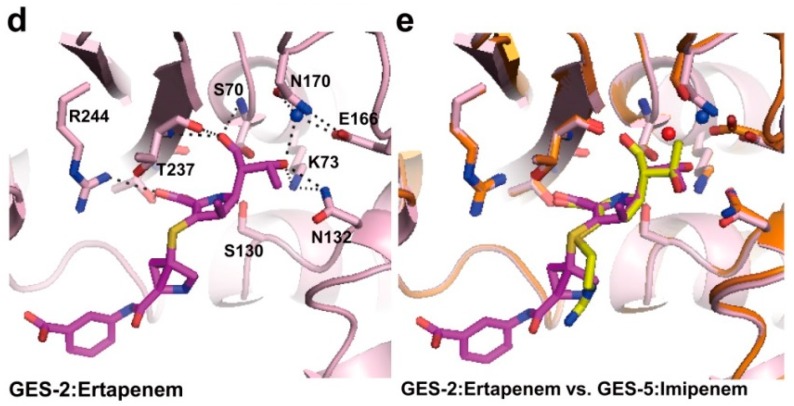
Imipenem acyl-enzyme intermediate complexes. (**a**) The active site of GES-1 (PDB entry 4GOG, green) with the bound imipenem (cyan) is shown; (**b**) The active site of GES-5 (PDB entry 4H8R, orange) with the bound imipenem (yellow) is shown; (**c**) Superposition of active sites of GES-1:imipenem and GES-5:imipenem is shown; (**d**) The active site of GES-2 (PDB entry 4QU3, light pink) with the bound ertapenem (magenta) is shown; (**e**) Superposition of active sites of GES-2:ertapenem and GES-5:imipenem is shown. The residues (S70, K73, S130, N132, E166, G170/S170/N170, T237, and R244) in the active-site cleft are shown as sticks. The hydrogen bond interactions are shown as dashed black lines. The partially occupied water molecules are shown as red and blue spheres. Superpositions were performed using SSM Superpose [53] to align the complete chains. These figures were prepared using *PyMOL* [54] and data adapted from Smith *et al.* [36] and Stewart *et al.* [57].

The crystal structures of imipenem complexes of GES-1 and GES-5 have shown that better coordination of the deacylating water molecule for effective deacylation is made by one amino-acid substitution (Gly170Ser) between GES-1 and GES-5 (Figure 3) [36]. In most class A carbapenemases, this residue is Asn170 which exists in the active site, while GES-1 has Gly170 in the active site and it is replaced by Ser170 in GES-5 (Figure 3a,b). A previous report [56] revealed that the catalytic efficiency of GES-5 for imipenem (0.295 µM^−1^·S^−1^) was 100-fold higher than that of GES-1 (0.003 µM^−1^·S^−1^). This result means that GES-5 has high carbapenem-hydrolyzing activity, while GES-1 has very poor activity for carbapenem. Superimposition of imipenem complexes of GES-1 and GES-5 showed almost perfect overlap of the two structures; however, the conformation of the GES-5 active site by Ser170 residue moved toward the active site Ser70 by ~1 Å compared with GES-1 (Figure 3c) [36]. In GES-5, Glu166 made new hydrogen bond interaction with one of the conformations of the Ser170 side chain and the hydrolytic water molecule located closely to Ser70 and Ser170 (Figure 3b). Thus, the presence of the hydrogen bond interaction between Ser170 and Glu166 may play a crucial effect on improving carbapenemase activity [36]. In addition, the crystal structure of GES-2 in complex with ertapenem has been determined recently [57]. The GES-2 enzyme also differs by only one amino acid (Asn170) at position 170 compared with GES-1 and GES-5 enzymes. Superimposition of ertapenem complex of GES-2 and imipenem complex of GES-5 showed that two carbapenems are bound in a very similar way in the two enzymes, with the exception of the 6α-hydroxyethyl group of carbapenem (Figure 3b,d) [57]. The C8 hydroxyl of 6α-1*R*-hydroxyethyl group of ertapenem formed the hydrogen bond with Asn132 in GES-2. However, when the C8 hydroxyl of 6α-1*R*-hydroxyethyl group of imipenem formed the hydrogen bond with Asn132 in GES-5, the hydroxyethyl group was rotated by 120° (Figure 3b,d) [57]. This rotation may lead to the conformational rearrangement of the 6α-hydroxyethyl group to avert severe steric hindrance with the side chain of Asn170 in GES-2. Another difference between GES-2 and GES-5 is the location of deacylation water in the active site. In GES-5, the hydrolytic water molecule is located closely to Ser70 and Ser170; however, the hydrolytic water in GES-2 is positioned between Glu166 and Asn170 (Figure 3e) [57]. Thus, this result suggested that the hydrogen bonding interaction between the deacylation water and the side chains of Glu166 and Asn170 in GES-2 may make a significantly more stable water binding site compared to the equivalent binding site in GES-5.

**Figure 4:**
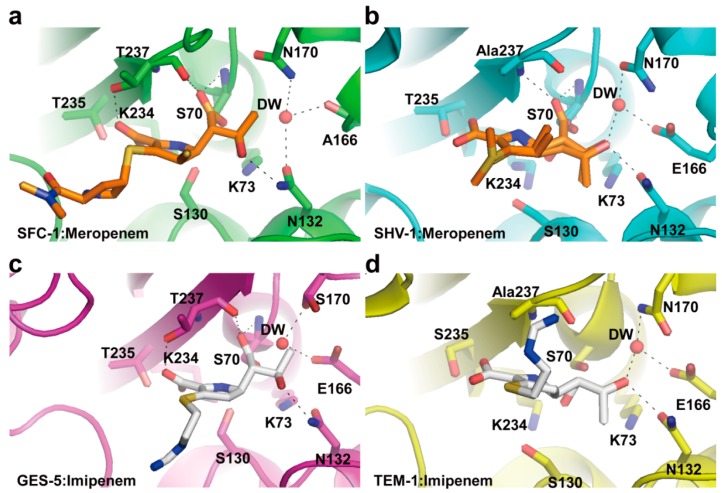
Comparison between carbapenem acyl-enzymes of class A carbapenemases (SFC-1 and GES-5) and non-carbapenemases (SHV-1 and TEM-1). (**a**) SFC-1 (E166A mutant):meropenem (PDB entry 4EV4, green); (**b**) SHV-1:meropenem (PDB entry 2ZD8, cyan); (**c**) GES-5:imipenem (PDB entry 4H8R, magenta); and (**d**) TEM-1:imipenem (PDB entry 1BT525, yellow). The residues (S70, K73, S130, N132, E166/A166, N170/S170, T235, and T237) in the active-site cleft are shown as sticks. Meropenem and imipenem carbon atoms are rendered in orange and white, respectively. Hydrogen bonds involving the deacylating water molecule (DW, red sphere), the acyl-enzyme carbonyl group, the carbapenem C3 carboxylate, the 6α-1*R*-hydroxyethyl group of carbapenem, and Asn132 NH_2_ are indicated by dashed black lines. These figures were prepared using *PyMOL* [54] and data adapted from Fonseca *et al.* [37].

The crystal structures of SFC-1 (E166A mutant) in complex with meropenem and GES-5 in complex with imipenem have been determined [36,37]. To understand the carbapenem-hydrolyzing mechanism of class A carbapenemases, they were compared with equivalent complexes for carbapenem-inhibited enzymes (non-carbapenemases) such as the meropenem acyl-enzyme complex of SHV-1 [58] and the imipenem acyl-enzyme complex of TEM-1 [59]. Because the process of productive deacylation of the acyl-enzyme intermediate is most likely accelerated in class A carbapenemases, the structural comparison is focused on the environment of the deacylating water (DW). Structural comparisons between the SFC-1(E166A mutant):meropenem, GES-5:imipenem complexes, the SHV-1:meropenem, and TEM-1:imipenem complexes revealed that their interaction differences were made by the DW molecule (Figure 4). In the SHV-1:meropenem and TEM-1:imipenem complexes, the DW in active site formed hydrogen bonds to Glu166, Asn170, and the C8 hydroxyl of 6α-1*R*-hydroxyethyl group of carbapenems (meropenem and imipenem) which formed hydrogen bond to Asn132 (Figure 4b,d). However, the meropenem complex of SFC-1(E166A mutant) and the imipenem complex of GES-5 showed that the DW in active site lost interaction with the C8 hydroxyl group of 6α-1*R*-hydroxyethyl group of carbapenems and newly formed hydrogen bond to Asn132 (Figure 4a,c). It was proposed that the DW is deactivated by interaction with the 6α-1*R*-hydroxyethyl group of carbapenem. Therefore, the difference of carbapenemase activity between carbapenemases (SFC-1 and GES-5) and non-carbapenemases (SHV-1 and TEM-1) may be caused by a different binding mode of the 6α-1*R*-hydroxyethyl group of carbapenems. In SFC-1 and GES-5, the C8 hydroxyl group of the 6α-1*R*-hydroxyethyl group lost interaction with the DW and instead interacts with Asn132, by which the DW preserves its activity.

**Figure 5:**
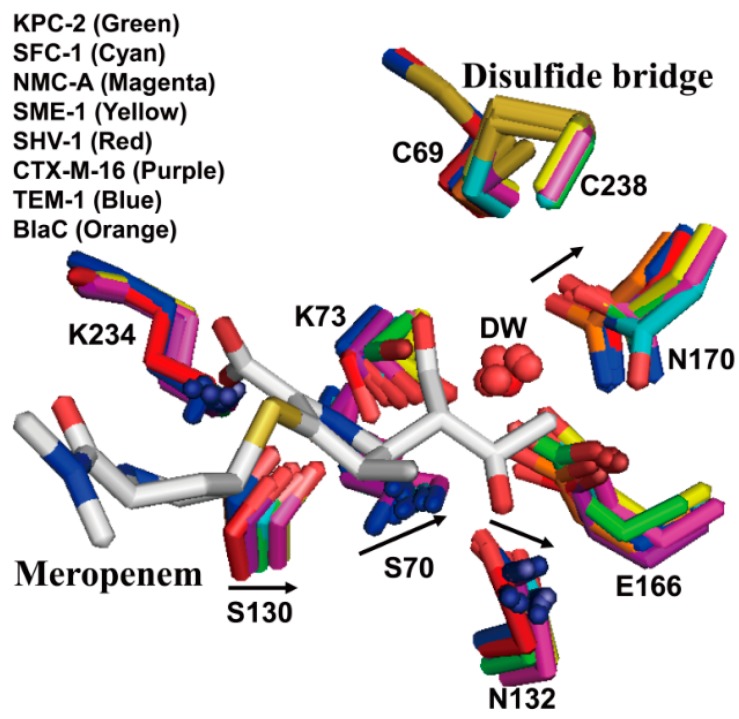
Superposition of active sites of class A carbapenemases (KPC-2 (PDB entry 2OV5, green), SFC-1 (E166A mutant; PDB entry 4EQI, cyan), NMC-A (PDB entry 1BUE, magenta), and SME-1 (PDB entry 1DY6, yellow)) and non-carbapenemases (SHV-1 (PDB entry 1SHV, red), CTX-M-16 (PDB entry 1YLW, purple), TEM-1 (PDB entry 1ZG4, blue), and BlaC (PDB entry 2GDN, orange)). Meropenem, as bound in the SFC-1 (E166A mutant) acyl-enzyme complex structure, is shown in white. The residues (C69, S70, K73, S130, N132, E166, N170, K234, and C238) in the active-site cleft are shown as sticks. Arrows denote shifts in positions of labeled residues in class A carbapenemases. There is a disulfide bridge between C69 and C238 in only class A carbapenemases. Superpositions were performed using SSM Superpose [53] to align the complete chains. This figure was prepared using *PyMOL* [54] and data adapted from Fonseca *et al.* [37].

In KPC-2, alteration of four residues (Ser70, Ser130, Asn132, and Asn170) of carbapenemases (KPC-2 [38], NMC-A [40], and SME-1 [39]) compared with non-carbapenemase (SHV-1 [60] and TEM-1 [61]) has been observed [38]. The shift of key residues such as Ser70, Ser130, Asn132, and Asn170 in SFC-1 compared with non-carbapenemases (SHV-1 [60], CTX-M-16 [62], BlaC [63], and TEM-1 [61]) has also been revealed [37]. A less buried position of side chain of Ser70 in the active site of carbapenemases allows easier access of the bulkier substrates (Figure 5). The shifts of Asn132 and Asn170 also increased the space adjacent to the water pocket (Figure 5). Ke *et al.* [38] explained that these structural differences may arise from a distinct conformation of the protein backbone owing to the disulfide bridge (Cys69–Cys238) in class A carbapenemases. It has been suggested that the movement of key residues in the active site of carbapenemases may enlarge the active site and permit the access of carbapenem to the active site by rotating the 6α-1*R*-hydroxyethyl group of the carbapenem [37,38].

### 2.3. Class C Carbapenemases

Class C β-lactamases pose therapeutic problems because they can confer resistance to cephamycins (cefoxitin and cefotetan), penicillins, cephalosporins, and β-lactam-β-lactamase inhibitor combinations and are not significantly inhibited by clinically used β-lactamase inhibitors such as clavulanic acid. They are mainly chromosomal class C β-lactamases in several potential pathogens, such as *Acinetobacter* spp., *Aeromonas* spp., *Chromobacterium violaceum*, *Citrobacter* spp., *Enterobacter* spp., *Escherichia coli*, *Morganella* spp., *Proteus rettgeri*, *P. aeruginosa*, *Serratia* spp., and *Yersinia enterocolitica* [64,65]. In addition, the plasmid-encoded class C β-lactamases have been reported in *K. pneumoniae* (CMY-1, CMY-2, CMY-8, CMY-12, MOX-1, MOX-2, FOX-1, FOX-5, LAT-1, LAT-2, LAT-2b, ACT-1, MIR-1, ACC-1, ACT-3, DHA-2, and DHA-3), *Klebsiella oxytoca* (CMY-5 and FOX-3), *E. coli* (CMY-4, CMY-6, CMY-7, CMY-9, CMY-11, CMY-13, FOX-2, FOX-4, B1L-1, LAT-3, LAT-4, ACC-4, and DHA-6), *Salmonella enteritidis* (DHA-1), *Proteus mirabilis* (CMY-3, CMY-12, CMY-14, and CMY-15), *Salmonella senftenberg* (CMY-2b), *Enterobacter aerogenes* K9911729 (CMY-10), *Pantoea agglomerans* (ACT-9), *S. marcescens* (ACT-10), and *E. cloacae* (DHA-7) [8,9,10,11,64,66,67,68,69,70,71,72,73,74,75,76,77,78]. Compared with chromosomal enzymes, plasmid-encoded class C β-lactamases are more problematic because they are transmissible to other bacterial species and are often expressed in large amounts [79]. Recently, five class C carbapenemases (namely ACT-1, DHA-1, CMY-2, CMY-10, and ADC-68) have been reported [9,11,12,66]. ACT-1, DHA-1, CMY-2, and CMY-10 are plasmid-encoded class C β-lactamases that exhibit catalytic activity for imipenem [8,80]. In particular, CMY-10 is the first reported carbapenemase among plasmidic class C β-lactamases [80], and also a class C extended-spectrum β-lactamase which has extended substrate specificity to extended-spectrum cephalosporins [81,82]. The chromosomal AmpC genes in *Acinetobacter* spp. are designated as ADCs (*Acinetobacter*-derived cephalosporinases) [83,84]. ADCs can hydrolyze cephalosporins such as cefotaxime and ceftazidime but not cefepime or carbapenems. ADC-68 from *A. baumannii* D015 was the first reported enzyme among chromosomal class C β-lactamases to possess class C extended-spectrum β-lactamase and carbapenemase activities [12]. The catalytic efficiencies (*K*_cat_/*K*_m_) of ACT-1 [8], CMY-2 [8], CMY-10 [80], and ADC-68 [12] for imipenem were 0.007, 0.04, 0.14, and 0.17 µM^−1^·S^−1^, respectively.

### 2.4. Structural Components and Catalytic Mechanism of Class C Carbapenemases

**Figure 6:**
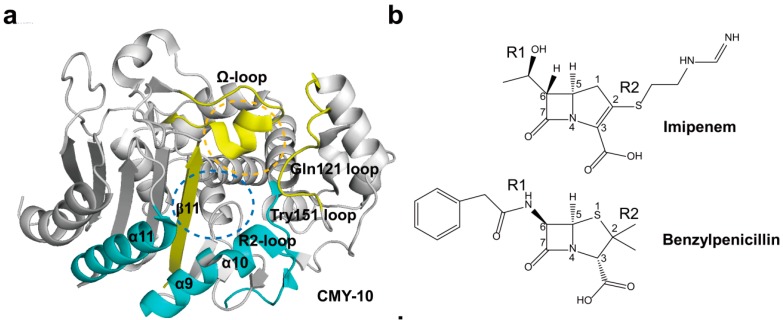

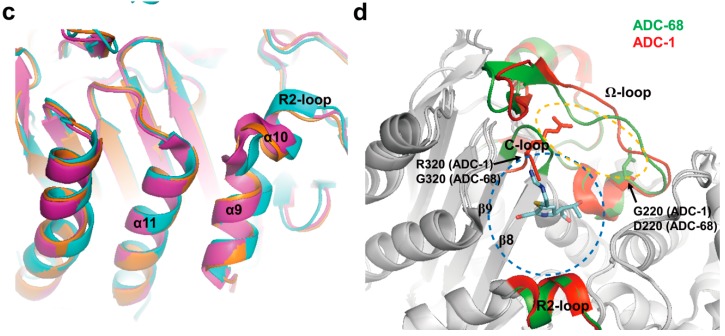
(**a**) Overall structure of CMY-10 (PDB entry 1ZKJ) is shown. The R1 subsite is surrounded by the Ω-loop, Gln121 loop, and β11 (in yellow). The R2 subsite is enclosed by the Tyr151 loop, α10 in the R2-loop, and α11 (in cyan). The R1 and R2 subsites are indicated as orange and blue dotted circles, respectively; (**b**) Schematic drawing of imipenem and benzylpenicillin is shown. The β-lactam nucleus is numbered. The R1 and R2 side chains located at the C6 and C2 positions of the β-lactam nucleus are labeled, respectively; (**c**) The displacement of α9 and α10 in CMY-10 is shown. CMY-10 (PDB entry 1ZKJ, cyan) was superposed with P99 β-lactamase (PDB entry 2BLT, orange) and GC1 β-lactamase (PDB entry 1GCE, magenta [89]). The R2-loop displays noticeable structural alterations: the R2-loop becomes flexible, and the shortened path of the connection loop between α10 and β11 induces the ~2.5 Å shift of α9 and α10 relative to the adjacent helix α11 in CMY-10 compared with both P99 and GC1 β-lactamases; (**d**) Superimposed complex of imipenem with ADC-68 and ADC-1. An AmpC complex with imipenem (PDB entry 1LL5) was superposed with ADC-1 (PDB entry 4NET) and ADC-68 (PDB entry 4QD4). ADC-68 and ADC-1 are represented as green and red ribbon diagrams, respectively. Imipenem is represented as cyan stick. C-loop (T318–F321) is positioned between β8 and β9. Ω-loop (G185–T229) is positioned between α6 and α8. R2-loop (E291–V309) is positioned between α9 and α10b. The R1 and R2 subsites are indicated as orange and blue dotted circles, respectively. R320 (ADC-1) and G320 (ADC-68) residues are located in C-loop and G220 (ADC-1) and D220 (ADC-68) residues are found in Ω-loop. Superpositions were performed using SSM Superpose [53] to align the complete chains. The structures of CMY-10 and ADC-68 were prepared using *PyMOL* [54] and data adapted from Kim *et al.* [80] and Jeon *et al.* [12], respectively.

Compared with class A β-lactamases, class C β-lactamases have larger active site cavities which may permit the binding with the bulky extended-spectrum cephalosporins. However, four important structural motifs in class A β-lactamases are also conserved in class C β-lactamases. The overall structures between class C β-lactamases are very similar and consisted of two main domains of which domain 1 has only α-helix and domain 2 comprises an α/β domain [85]. Their active-site motifs, Ser64-X-X-Lys67, Tyr150-X-Asn152, and Lys315-Thr316-Gly317, were conserved in all class C β-lactamases [86]. The active site lies in the center of the enzyme at the left edge of the five-stranded β-sheet with the reactive serine residue [85]. The active site can be divided into two subsites: R1 and R2 subsites. The R1 subsite refers to the region that accommodates the R1 side chain at C7 (C6) of the β-lactam nucleus in β-lactam antibiotics, and the R2 subsite represents the opposite region interacting with the right part of the β-lactam ring including the R2 side chain at C3 (C2) [80]. The R1 subsite is surrounded by the Ω-loop and the R2 subsite is enclosed by the R2-loop containing the α-10 and α-11 helices (Figure 6a) [80,85]. They act on acylation and deacylation steps. In the acylation step, the serine residue attacks the carbonyl carbon of the β-lactam ring to form an acyl-enzyme intermediate. In the next deacylation step, the acyl-enzyme adduct is attacked by a water molecule, releasing hydrolyzed antibiotics [86,87]. Crystal structures of class C carbapenemases, CMY-10 and ADC-68, have been determined [12,80].

The R1 side chain (the hydroxyethyl group at C6) of imipenem is much smaller than that of benzylpenicillin (Figure 6b). Thus, it is not likely that the small R1 side chain impedes the hydrolysis because there is no problem in the accommodation of the small side chain by class C enzymes. Instead, the long R2 side chain of imipenem is the main cause of the catalytic failure [5]. The crystallographic study of the CMY-10 has revealed that a three-amino-acid deletion in the R2-loop of CMY-10 significantly widens the R2 subsite, which accommodates the R2 side chains of β-lactams (Figure 6c) [80]. Based on this perspective, it was tested whether CMY-10 would hydrolyze imipenem. As expected, CMY-10 hydrolyzed imipenem with considerable catalytic efficiency [5,80]. A modeling study of CMY-2 and ACT-1 also revealed that their large R2 subsites might improve their accommodation of imipenem inside the catalytic pocket [8]. These results suggested that structural differences in the R2 subsite may have an effect on carbapenem hydrolytic activity.

Compared with ADC-1 (a non-carbapenemase) [88], ADC-68 shares 98% sequence identity and has seven amino-acid substitutions [12]. Although the overall structures of ADC-68 and ADC-1 were conserved, noticeable structural differences were found in the Ω-loop and C-loop (Figure 6d). The loop between β8- and β9-strands was named a C-loop because of the structural possibility of the importance of this region in acquiring carbapenemase activity. In particular, the Gly320 located in the C-loop and Asp220 found in the Ω-loop contributed to the major structural differences between ADC-68 and ADC-1. The C-loop is located just beneath the central part of the Ω-loop (Figure 6d). Accordingly, the conformation of the C-loop is directly related to that of the adjacent Ω-loop. Compared with Arg320 in ADC-1, ADC-68 has the much smaller Gly320 residue in the C-loop. The bulkier Arg320 residue of the C-loop in the ADC-1 structure simultaneously interfered with the stable formation of both the C-loop and the central Ω-loop because of steric hindrance. When the central Ω-loop was activated in ADC-1, it pushed the C-loop into the R2 subsite, also disrupting part of the β8-strand (Figure 6d). However, ADC-68 crystal structure had an intact C-loop compared with the bulged-in C-loop of ADC-1. When the imipenem-bound AmpC structure was superimposed with ADC-68 and ADC-1 (the bulged-in C-loop), the R2 side chain of imipenem was exactly superimposed on the bulged-in C-loop of ADC-1 and imipenem could not be accommodated in the R2 subsite of ADC-1. However, in ADC-68, no steric hindrance was observed (Figure 6d). ADC-68 also has a three-amino-acid deletion in the R2-loop as in CMY-10, which causes a widening of the R2 binding pocket by forming a wide-open conformation of the R2-loop. These results suggested that the stable open conformation of the R2 subsite of ADC-68 could help bind the carbapenems [12].

### 2.5. Class D Carbapenemases

Class D β-lactamases were referred to as OXAs (oxacillinases) because they commonly hydrolyze the isoxazolylpenicillin oxacillin much faster than benzylpenicillin [90]. These numbers were verified by their unique amino acid sequence at the Lahey website (http://www.lahey.org/Studies/), where the complete list of OXA β-lactamases can be found. At present, the OXA type enzymes include more than 400 enzymes. Some variants among them actually possess carbapenemase activity. Based on their amino acid sequence, class D carbapenemases are recently reclassified into 12 subgroups: OXA-23, OXA-24/40, OXA-48, OXA-51, OXA-58, OXA-134a, OXA-143, OXA-211, OXA-213, OXA-214, OXA-229, and OXA-235 [91]. An original sequencing error in OXA-24 made it now OXA-40 (see the Lahey website) and thus OXA-24 (or OXA-40) was re-designated as OXA-24/40. The sequence identities between members of each subgroup are more than 90%, whereas the identities between enzymes that belong to different subgroups are less than 70% [91,92]. The first subgroup was the OXA-23 subgroup. The OXA-23 enzyme was first identified in an *A. baumannii* isolate collected in the UK in 1985 [93]. Since then, the 18 variants (alleles) of the *bla*_OXA-23_ gene have been identified. The second subgroup was the OXA-24/40 subgroup. The OXA-24/40 was identified in isolates in Spain in 1997 [94] and the six variants of the *bla*_OXA-24/40_ gene have been identified. The third subgroup was OXA-48 subgroup. The OXA-48 was identified in *K. pneumoniae* in Turkey in 2003 [95] and the 10 variants of the *bla*_OXA-48_ gene have been identified. The fourth subgroup was the OXA-51 subgroup and this subgroup is the largest subgroup among OXA-type β-lactamases. The OXA-51 enzyme was first identified in *A. baumannii* isolates from Argentina isolated in 1996 [96] and the 94 variants of the *bla*_OXA-51_ gene have been identified. The fifth subgroup was the OXA-58 subgroup. The OXA-58 enzyme was identified in a multidrug-resistant *A. baumannii* clinical isolate in France in 2003 [97] and the three variants of the *bla*_OXA-58_ gene have been identified. The sixth subgroup was the OXA-134a subgroup. The OXA-134a enzyme was identified in an isolated *Acinetobacter lwoffii* [98] and the six variants of the *bla*_OXA-134a_ gene have been identified. The seventh subgroup was the OXA-143 subgroup. OXA-143 enzyme was identified in an *A. lwoffii* isolate in Brazil in 2004 [99] and the four variants of the *bla*_OXA-143_ gene have been identified. Recent studies aiming to identify the naturally occurring OXA-type enzymes of other *Acinetobacter* species have successfully identified a number of new enzyme subgroups [91]. These new subgroups included the OXA-211 enzyme subgroup from *Acinetobacter johnsonii* [100], the OXA-213 enzyme subgroup from *Acinetobacter calcoaceticus* [100], the OXA-214 enzyme subgroup from *Acinetobacter haemolyticus* [99], the OXA-229 enzyme subgroup from *Acinetobacter bereziniae* [101], and the OXA-235 enzyme subgroup from *Acinetobacter schindleri* [102].

### 2.6. Structural Components and Catalytic Mechanism of Class D Carbapenemases

Like cases of class A and C β-lactamases, class D β-lactamases possess four important structural motifs. Crystal structures of class D carbapenemases, OXA-23, OXA-24/40, OXA-48, OXA-58, and OXA-146 [103,104,105,106,107,108], and class D non-carbapenemases, OXA-1 from *E. coli*, OXA-10 from *P. aeruginosa*, and OXA-13 from *P. aeruginosa* [109,110,111], have been determined. The catalytic efficiencies (*K*_cat_/*K*_m_) of OXA-23 [104], OXA-24/40 [112], OXA-48 [113], and OXA-58 [114] for imipenem were 0.073, 0.015, 0.37, and 0.169 µM^−1^·S^−1^, respectively. OXA-48 is the most efficient class D carbapenemase for imipenem compared with other class D carbapenemases. Their overall structures are similar to those of other class D β-lactamases (OXA-1, OXA-10, and OXA-13) and consists of two domains of which one domain comprises helixes and another domain has a mixed α/β domain including a central six-stranded antiparallel β-sheet. Their active-site motifs, Ser70-X-X-Lys73, Ser118-X-Val/Ile120, and Lys216-Thr/Ser217-Gly218 (Ser70 according to the DBL (class D β-lactamase) numbering) [115], are conserved and the active site lies between the interface of the β-sheet and the helical subdomain. Two conserved motifs, Tyr/Phe144-Gly145-Asn146 and the Trp232-X-X-Gly235, have no analogues in either class A or class C β-lactamases [92]. The enzymatic reaction of Class D β-lactamases including class D carbapenemases also include acylation and deacylation steps. In particular, acylation and deacylation steps are facilitated by the conserved lysine residue, which is *N*-carboxylated in a posttranslational step [116,117]. This is the unique feature of class D β-lactamases. In class D carbapenemases (OXA-23, OXA-48, and OXA-58), each lysine located in the first motif is carboxylated at neutral pH [104].

**Figure 7:**
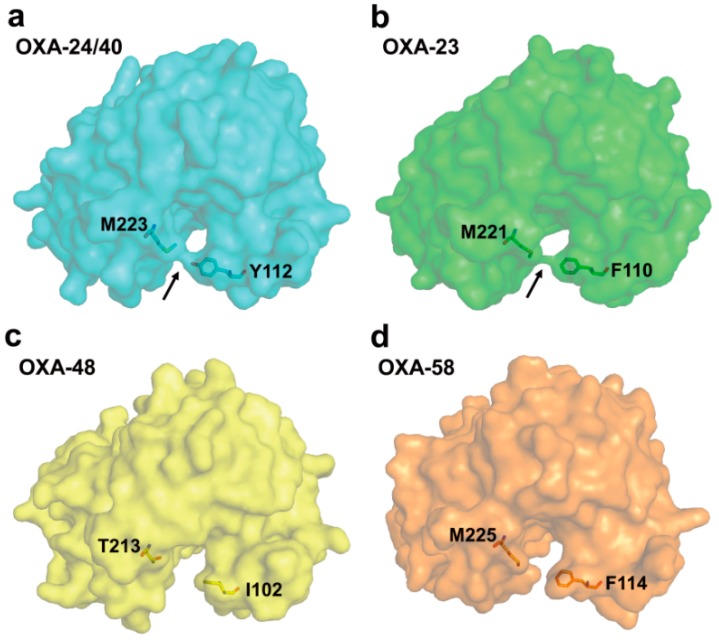
Molecular surface representations of the class D carbapenemases. (**a**) OXA-24/40 (PDB entry 2JC7, cyan); (**b**) OXA-23 (PDB entry 4JF6, green); (**c**) OXA-48 (PDB entry 3HBR, yellow); and (**d**) OXA-58 (PDB entry 4OH0, orange). The four molecules are shown in the same relative orientation, and the active site exists at the bottom center of each figure. The hydrophobic bridge (arrow) is shown in OXA-23 and OXA-24/40. A lack of a similar bridge in OXA-48 and OXA-58 is evident. The residues related to the hydrophobic bridge are indicated as sticks. These figures were prepared using *PyMOL* [54] and data adapted from Smith *et al.* [103].

The crystal structure of OXA-24/40 has been first reported in [108]. The tunnel-like entrance to the active site in OXA-24/40 was established by a hydrophobic barrier that was formed through the specific arrangement of the Tyr112 and Met223 side chains (Figure 7a). The tunnel may easily permit the small size of the hydroxyethyl group of carbapenems to have access to the active site, whereas it may restrict the access of antibiotics with bulkier group at position C6 of the β-lactam ring such as oxacillin, cloxacillin, and methicillin. Santillana *et al.* [108] suggested that the hydrophobic bridge is likely to contribute to a very strong binding affinity of OXA-24/40 for carbapenem. In addition, the hydrophobic bridge to the active site in OXA-23 is formed by the side chains of Phe110 and Met221 (Figure 7b) [104]. However, the active site in OXA-48 cannot form the hydrophobic bridge because Tyr112 and Met223 in OXA-24/40 are replaced by the Ile102 and Thr213 in OXA-48, respectively (Figure 7c) [107]. In OXA-58, there is no the hydrophobic bridge in the active site, even though the corresponding residues (Phe114 and Met225 in OXA-58) are intact (Figure 7d) [103]. Compared with OXA-24/40, OXA-48 and OXA-58 have a differently shaped active site. Moreover, OXA-48 and OXA-58 have a higher catalytic efficiency (*K*_cat_/*K*_m_) for imipenem than OXA-23 and OXA-24/40. These observations suggested that the hydrophobic bridge may not be the sole structural determinant for more efficient deacylation of carbapenems in class D carbapenemases [103].

**Figure 8:**
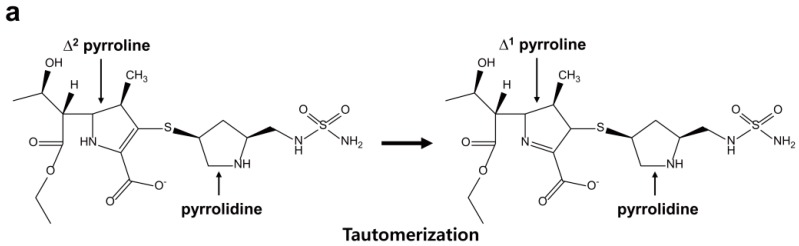

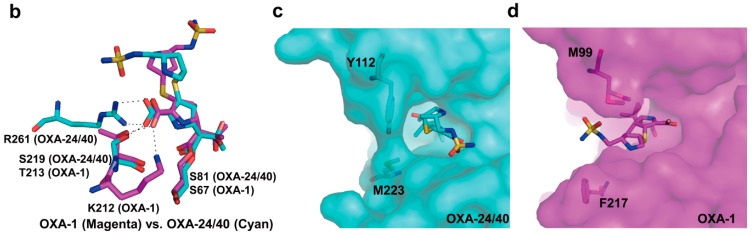
(**a**) Pyrroline tautomerization after doripenem acylation of some β-lactamase enzymes such as OXA-1; (**b**) The superposition of OXA-1:doripenem (PDB entry 3ISG, magenta) and OXA-24/40 (K84D mutant):doripenem (PDB entry 3PAE, cyan) active sites is shown. Residues (S81, S219, and R261) from OXA-24/40 K84D and residues (S67, K212, and T213) from OXA-1 are shown. The hydrogen bond interactions are shown as dashed black lines; (**c**) Doripenem and two active site residues of OXA-24/40 (K84D mutant) are shown in cyan; (**d**) Doripenem and two active site residues of OXA-1 are shown in magenta. Superpositions were performed using SSM Superpose [53] to align the complete chains. These figures were prepared using *PyMOL* [54] and data adapted from Schneider *et al.* [106].

The crystal structures of two OXA-24/40 mutants (Lys84Asp and Val130Asp) in complex with doripenem have been determined [106]. To understand the carbapenem-hydrolyzing mechanism of class D carbapenemase, two variants in complex with doripenem were compared with OXA-1 in complex with doripenem. Compared with the pyrroline ring of doripenem in OXA-24/40, that of doripenem in OXA-1 was moved laterally by ~0.7 Å (Figure 8b). In OXA-1:doripenem complex, the side chains of Lys212 and Thr213 form a salt bridge and hydrogen bond with the carboxylate of doripenem, respectively (Figure 8b). In OXA-24/40:doripenem complex, the side chains of Ser219 and Arg261 interact with the carboxylate of doripenem and provide stabilizing side chain interactions (Figure 8b). In addition, the sulfur atom which bridges the pyrroline and pyrrolidine rings is angled above the former by ~30° in the OXA-1 compared with OXA-24/40:doripenem complex (Figure 8b). Moreover, the hydrophobic bridge formed by Tyr112 and Met223 in OXA-24/40 caused drastically different binding conformation compared with OXA-1:doripenem complex (Figure 8c,d). These structural differences revealed that the pyrroline ring of carbapenems in OXA-1 and OXA-24/40 is present in two alternative tautomeric forms, of which the Δ^1^ tautomeric form has the pyrroline ring of an imine form in OXA-1 and the Δ^2^ tautomeric form has the pyrroline ring of an enamine form in OXA-24/40 (Figure 8a). Easton and Knowles [118] suggested that hydrolysis of the β-lactam ring could lead to tautomerization of the pyrroline ring and the Δ^2^ tautomeric form was thought to be the catalytically competent isomer. According to these results, Schneider *et al.* [106] suggested that the tautomeric form in OXA-24/40 remain in the Δ^2^ tautomeric form rather than the Δ^1^ tautomeric form as observed in OXA-1 owing to the planar orientation of this sulfur of doripenem.

**Figure 9:**
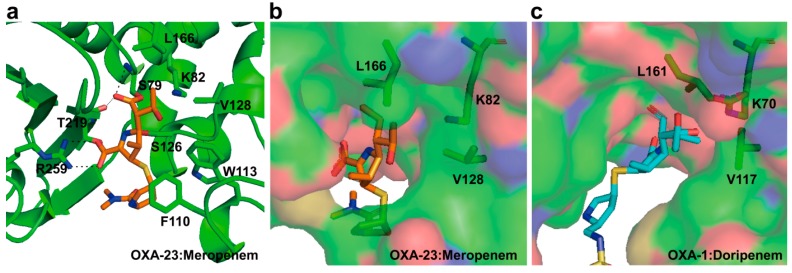
(**a**) Active site of the OXA-23 in complex with meropenem (PDB entry 4JF4) is shown. The residues (S79, K82, F110, W113, S126, V128, L166, T219, and R259) in the active-site cleft are shown as green sticks. Meropenem is represented as orange stick. The hydrogen bond interactions between meropenem and residues in the active site in OXA-23 are shown as dashed black lines; (**b**) Molecular surface representation of OXA-23 in complex with meropenem is shown. The side chains of K82, V128, and L166 in OXA-23 are shown in sticks; (**c**) Molecular surface representation of OXA-1 in complex with doripenem (PDB entry 3ISG) is shown. Doripenem is represented as cyan stick. The side chains of K70, V117, and L161 in OXA-1 are shown in sticks. These figures were prepared using *PyMOL* [54] and data adapted from Smith *et al.* [104].

The crystal structure of wild type OXA-23 in complex with meropenem at pH 4.1 has been first determined in [104]. Meropenem is covalently bound to the Oγ atom of Ser79 (nucleophile; OXA-23 numbering) and anchored by hydrogen bond interactions along with a number of hydrophobic packing contacts, and two residues (Phe110 and Trp113) at α3–α4 loop provide aromatic packing contacts with the pyrrolidine ring of meropenem (Figure 9a). The oxygen atom of 6α-1*R*-hydroxyethyl group of meropenem is directed toward the side chains of Val128 and Leu166, and is directed away from the active site Ser79 (Figure 9a). This feature was consistent with structural data in the OXA-24/40 (K84D mutant):doripenem complex [105]. The side chain of Leu166 in OXA-23:meropenem complex showed a conformational change compared with OXA-1:doripenem complex. In OXA-1:doripenem complex, the side chain of Leu161 (equivalent to Leu166 in OXA-23) makes a hydrophobic interaction with Val117 (equivalent to Val128 in OXA-23) and it may restrict the access of the hydrolytic water into the active site (Figure 9c), while Leu166 in the OXA-23:meropenem swings away from the 6α-1*R*-hydroxyethyl group and it may create a space to access the hydrolytic water into the active site (Figure 9b). According to these results, Smith *et al.* [104] proposed that the motion of Leu166 in OXA-23 play a critical role in allowing the access of the hydrolytic water to the *N*-carboxylated lysine (Lys82 in OXA-23 and Lys70 in OXA-1) to enable deacylation of the acyl-enzyme species in imparting carbapenemase activity. Schneider *et al.* [106] suggested that the hydrophobic bridge formed by Tyr112 and Met223 of OXA-24/40 would interfere with the formation of the Δ^1^-tautomer of carbapenem. In spite of forming the hydrophobic bridge between the chains of Phe110 and Met221 in OXA-23, tautomeric form in OXA-23:meropenem complex observed Δ^1^ tautomer (*S* stereoisomer), and it was different form with Δ^1^ tautomer (*R* stereoisomer) as seen in OXA-1:doripenem complex. Smith *et al.* [104] suggested that the hydrophobic tunnel may have less impact on forcing carbapenem substrate to adopt a Δ^2^ tautomer and a specific orientation of the 6α-1*R*-hydroxyethyl group.

**Figure 10:**
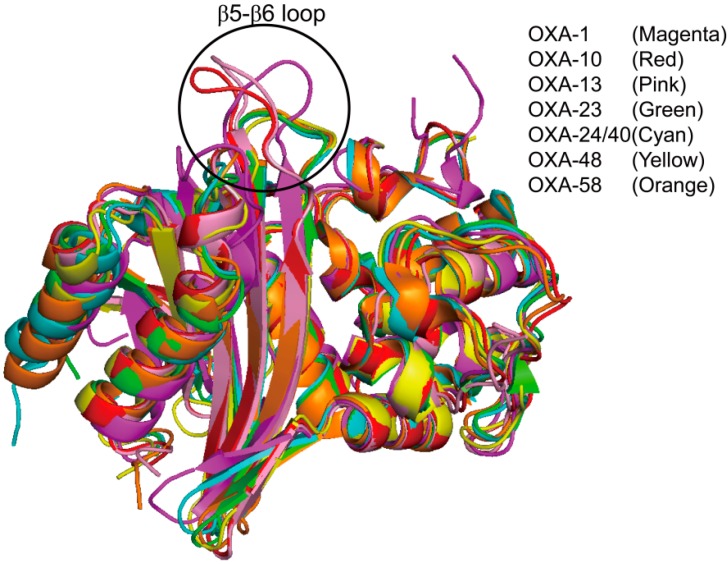
Comparison of the structural features of class D carbapenemases (OXA-23 (PDB entry 4JF6, green), OXA-24/40 (PDB entry 2JC7, cyan), OXA-48 (PDB entry 3HBR, yellow), and OXA-58 (PDB entry 4OH0, orange)) with the class D non-carbapenemases (OXA-1 (PDB entry 1M6K, magenta), OXA-10 (PDB entry 1FOF, red), and OXA-13 (PDB entry 1H8Z, pink)), showing the significant differences in orientation and length of the β5–β6 loop. The β5–β6 loops, connecting β5- and β6-strands, are indicated by the black circle. Superpositions were performed using SSM Superpose [53] to align the complete chains. These figures were prepared using *PyMOL* [54] and data adapted from De Luca *et al.* [119].

The overall structure of OXA-48 is very similar to those of OXA-1, OXA-10, and OXA-13 (non-carbapenemases). However, a structural difference between OXA-48 and non-carbapenemases appeared in the loop (β5–β6 loop) connecting β5- and β6-strands, which may vary in length and orientation. The length of the β5–β6 loop in OXA-48 is shorter than those of β5–β6 loops in OXA-1, OXA-10, and OXA-13 (Figure 10). This loop is located inwards the active site in OXA-48 and forms a narrow active-site cleft. This feature also existed in other carbapenemases (OXA-23, OXA-24/40, and OXA-58) (Figure 10). Docquier *et al.* [107] hypothesized that the short-loop connecting β5- and β6-strands plays a potential role in conferring the carbapenemase activity of the OXA-48 enzyme. De Luca and colleagues have performed direct evolution study on the non-carbapenemase (OXA-10) using three OXA-10 loop variants (OXA-10loop23, OXA-10loop24/40, and OXA-10loop48) which substituted the β5–β6 loop of the OXA-10 with the structurally equivalent loops of three class D carbapenemases (OXA-23, OXA-24/40, and OXA-48) [119]. Crystal structures and kinetic data revealed that although OXA-10loop24/40 and OXA-10loop48 did not show significant changes in the molecular fold of the enzyme except for the β5–β6 loop, three OXA-10 loop variants showed significant carbapenemase activity for imipenem. Taken together, the authors suggested that the β5–β6 loop in class D carbapenemases play a crucial role in the carbapenemase activity.

**Figure 11:**
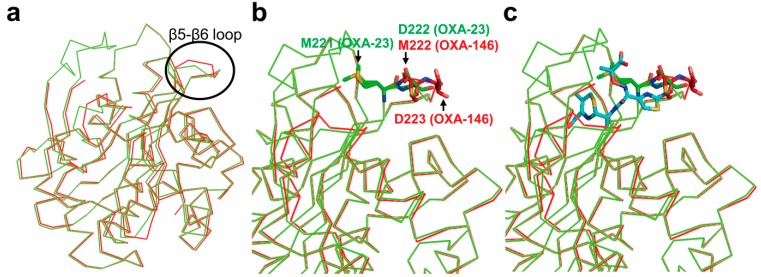
Comparison of OXA-23 (PDB entry 4K0X, green) and OXA-146 (PDB entry 4K0W, red) structures. (**a**) Overlaid structure showing the position of the β5–β6 loop deviation (black circle) in the context of the full structures of OXA-23 and OXA-146; (**b**) Overlaid structure of OXA-23 and OXA-146 is shown. Residues (M221 and D222, green) in OXA-23 and residues (M222 and D223, red) in OXA-146 are shown as sticks; (**c**) Overlaid structure of OXA-23 and OXA-146 after alignment with the β-lactam sensor protein BlaR1 with ceftazidime bound as an acyl intermediate (PDB entry 1XKZ). The ceftazidime serine acyl moiety is shown in cyan, and the rest of the BlaR1 protein is not shown. Superpositions were performed using SSM Superpose [53] to align the complete chains. These figures were prepared using *PyMOL* [54] and data adapted from Kaitany *et al.* [105].

The amino acid of OXA-146 differs from OXA-23 by only the insertion of a single alanine residue (Ala220, OXA-146 numbering). The insertion occurs in the β5–β6 loop involved in the acquisition of carbapenemase activity. Surprisingly, a kinetic study has revealed that OXA-146 hydrolyzed extended-spectrum cephalosporins (e.g., ceftazidime) as well as carbapenems [105]. In the case of the doripenem, OXA-146 showed the same efficacy with the parental OXA-23 enzyme. The overall structures of OXA-146 and OXA-23 are nearly identical, but, the structural difference between the two structures took place in the β5–β6 loop (Figure 11a). The second alanine (Ala221) in OXA-146 is positioned at Met221 in OXA-23. Therefore, there is the movement of the Met222 (Met 221 in OXA-23) residue into the β5-β6 loop of OXA-146 and no hydrophobic bridge in the active site (Figure 11b). Throughout a model of ceftazidime bound in the active site, Kaitany *et al.* [105] described that the structural alteration caused by a single insertion in β5–β6 loop is likely to relieve steric clashes between the bulky R1 side chain of ceftazidime and OXA-23 (Figure 11c). The authors suggested that the structural variation in OXA-146 may lead to an increased activity for extended-spectrum cephalosporins.

## 3. Metallo-Carbapenemases: Zinc-Dependent Class B

### 3.1. Class B Carbapenemases

Unlike the serine dependent β-lactamases (classes A, C, and D), class B β-lactamases are metallo-β-lactamases (MBLs) that require zinc or another heavy metal for catalysis. MBLs have a broad substrate spectrum and can catalyze the hydrolysis of virtually all β-lactam antibiotics including carbapenems except for monobactams [120]. They are not inhibited by mechanism-based inhibitors such as clavulanate, sulbactam, tazobactam, or NXL-104, whereas they are inactivated by metal chelators such as EDTA (ethylene diamine tetraacetic acid) [48,121,122]. MBLs were initially discovered over 40 years ago and have been identified as acquired enzymes since the early 1990s, either in *Pseudomonas* or *Enterobacteriaceae* [123]. The most common families of acquired class B MBLs identified in *Enterobacteriaceae* include the VIM and IMP groups, together with the emerging NDM group [123,124,125]. IMP-type carbapenemases including 48 IMP variants were identified in a series of clinically important Gram-negative bacilli, such as *Enterobacteriaceae*, *Pseudomonas*, and *Acinetobacter* [34]. IMP-1 was found in an *S*. *marcescens* isolate in Japan in 1991 [126]. VIM-type carbapenemases including 41 VIM variants were identified in *Enterobacteriaceae* [34]. VIM-1 was first identified in Italy in 1997 [127,128], and VIM-2 was then reported in France in a *P. aeruginosa* isolate dating from 1996 [129]. One of the most clinically significant carbapenemases, NDM-1 (New Delhi metallo-β-lactamase), was first detected in 2008 in *K. pneumonia* and *E. coli* in a patient returning to Sweden from India and has been subsequently shown to be present in bacterial isolates in a number of countries worldwide [125]. So far, 15 NDM variants have been assigned [27]. As compared with NDM-1, NDM-4, NDM-5, and NDM-7 variants possess increased activity towards carbapenems [130,131,132,133]. The GIM-1 was first identified in a *P. aeruginosa* isolate from Germany [134] and the KHM-1 was identified in a *Citrobacter freundii* clinical isolate from Japan [135]. The other MBLs, SPM-1 [136], SIM-1 [137], DIM-1 [138], TMB-1 [139], and AIM-1 [140], were identified in *Pseudomonas* or *Acinetobacter*.

### 3.2. Structural Components and Catalytic Mechanism of Class B Carbapenemases

Based on primary amino acid sequence homology and metal requirement, the MBLs are divided into three subclasses (B1, B2, and B3). The subclass B1 β-lactamases have a tightly coordinated Zn1 and a loosely coordinated Zn2, whereas the subclass B3 β-lactamases have two zinc ions with similar binding affinity. However, the subclass B2 β-lactamases have one tightly coordinated zinc ion (Zn2) that is sufficient for maximal enzymatic activity, and the binding of a zinc ion (Zn1) at another zinc-binding site reduces the enzymatic activity. The B1 and B3 subclasses have a broad substrate spectrum that includes penicillins, cephalosporins and carbapenems, whereas the B2 subclass has a narrow substrate spectrum that includes carbapenems.

The crystal structure of the mono-zinc form of BcII has been first determined in [141]. Thereafter, the crystal structures of other subclass B1 enzymes, including the di-zinc form of BcII from *Bacillus* *cereus* [142], BlaB from *Chryseobacterium* *meningosepticum* [143], CcrA from *Bacteroides* *fragilis* [144], IMP-1 from *S. marcescens* [145], VIM-2 from *P. aeruginosa* [146], VIM-4 from *P.aeruginosa* [147], VIM-7 from *P.* *aeruginosa* [148], NDM-1 from *K. pneumonia* [149,150], SPM-1 from *P.* *aeruginosa* [151], and GIM-1 from *P.* *aeruginosa* [152,153], have been determined. The crystal structures of the subclass B2 enzymes, such as CphA from *Aeromonas hydrophila* [154] and Sfh-I from *S.* *fonticola* [155], have also been determined. The crystal structures of subclass B3 enzymes, including L1 from *Stenotrophomonas* *maltophilia* [156], FEZ-1 from *Fluoribacter gormanii* [157], BJP-1 from *Bradyrhizobium japonicum* [158], and AIM-1 from *P.* *aeruginosa* [159], have been determined. The catalytic efficiencies (*K*_cat_/*K*_m_) of BcII [160], BlaB [161], CcrA [160], IMP-1 [162], VIM-2 [128], VIM-4 [147], VIM-7 [163], NDM-1 [129], SPM-1 [151], GIM-1 [134], CphA [154], Sfh-I [164], L1 [160], FEZ-1 [165], BJP-1 [166], and AIM-1 [159] for imipenem were 0.1, 0.95, 0.74, 1.2, 0.99, 23, 3.7, 0.21, 075, 0.1, 3.5, 0.64, 0.72, 0.2, 0.06, and 5.4 µM^−1^ S^−1^, respectively.

**Figure 12:**
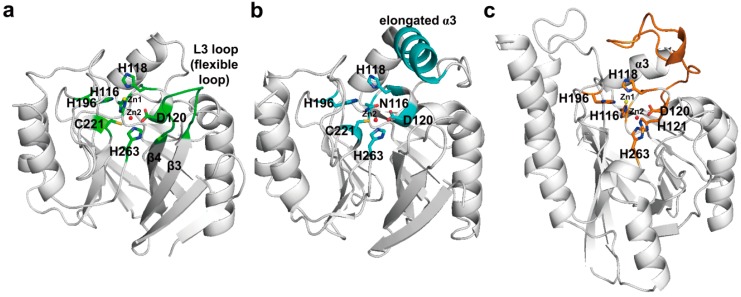
Ribbon representations of the three subclasses. (**a**) Overall structure of BcII (subclass B1, PDB entry 1BVT) from *B. cereus* is shown. The zinc-binding residues and flexible loop (L3 loop) are shown in green. The zinc-binding residues (H116-H118-H196 for Zn1 and D120-C221-H263 for Zn2) in the active-site cleft are shown as green sticks; (**b**) Overall structure of CphA (subclass B2, PDB entry 1X8G) from *A. hydrophila* is shown. The zinc-binding residues and elongated α3-helix are shown in cyan. The zinc-binding residues (N116-H118-H196 for Zn1 and D120-C221-H263 for Zn2) in the active-site cleft are shown as cyan sticks; (**c**) Overall structure of FEZ-1 (subclass B3, PDB entry 1K07) from *F. gormanii* is shown. The zinc-binding residues and the loop between α3-helix and β7-strand are shown in orange. The zinc-binding residues (H116-H118-H196 for Zn1 and D120-H121-H263 for Zn2) in the active-site cleft are shown as orange sticks. Zn1 and Zn2 are represented as yellow and red spheres, respectively. These figures were prepared using *PyMOL* [54] and data adapted from Garau *et al.* [154].

Although the MBLs exhibit a diverse range of sequences with as little as 25% identity between some enzymes, their overall structures are very similar and have a characteristic αβ/βα sandwich fold comprising two central β-sheets and five α-helices on the external faces [141,142,143,144,145,146,147,148,149,150,151,152,153,154,155,156,157,158,159]. The zinc-binding motifs in these scaffolds include six residues at the active site located at the external edge of the ββ sandwich. The zinc-binding motifs coordinate either one or two zinc ions that are central to the catalytic mechanism [119]. They can distinguish MBL subclasses as B1 (His116-His118-His196 and Asp120-Cys221-His263, class B numbering scheme [167]), B2 (Asn116-His118-His196 and Asp120-Cys221-His263), or B3 enzymes (His/Gly116-His118-His196 and Asp120-His121-His263) (Figure 12) [119]. In the case of B1 and B3 enzymes including two zinc-binding motifs, Zn1 is tetrahedrally coordinated by His116, His118, His196 (His/Gly116, His118, His196 in B3), and a water molecule or hydroxide (OH^−^) ion, and Zn2 has a trigonal-pyramidal coordination sphere which involves Asp120, Cys221, His263 (Asp120, His121, His263 in B3), and two water molecules. In the B1 and B3 subclasses, one water or OH^−^ ion serves as a ligand for both metal ions. It is believed that the hydroxide ion which is stabilized and activated by Zn1 and Zn2 performs a nucleophilic attack on the carbon of the carboxyl group of the β-lactam, leading to the formation of a transient, non-covalent tetrahedral intermediate that is stabilized by its interaction with the zinc ion [168,169,170]. In addition, Zn1 and Zn2 may involve protonation of the nitrogen of the cleaved β-lactam ring, and subsequent breakdown of the tetrahedral intermediate. In the case of B2 enzyme, Zn2 is bound to Asp120, Cys221, His263, and a solvent molecule, and these enzymes are active only in one zinc form (Zn2). However, it has been proposed that the nucleophilic hydroxide which is activated by the Asp120 and His118 residues, but not Zn2, attacks on the carbon of the carboxyl group of the β-lactam [154,171]. In addition, it has also been proposed that protonation of the nitrogen may occur through a water molecule bound by His118 and Asp120 residues or a water molecule bound to Zn2. Because MBLs are metalloenzymes, they are resistant to almost all conventional β-lactam inhibitors, but are inhibited by chelating agents such as EDTA and EGTA (ethylene glycol tetraacetic acid).

In the subclass B1 enzymes, they have the flexible L3 loop between β3- and β4-strands which possess hydrophobic side chains (Figure 12a). This loop is thought to have an important role in the binding of substrates and inhibitors. It closes over the bound substrate or inhibitor when the substrate or inhibitor diffuses into the active site [172]. NMR studies on CcrA has revealed that significant chemical shifts of L3 loop residues (flexible loop) are changed upon inhibitor binding, accompanied by decreased motion [173]. The subclass B2 CphA possesses an elongated α3-helix (residues Arg140-Leu161) adjacent to the active site groove which provides a hydrophobic face that contributes to the binding of carbapenem substrates (Figure 12b) [154]. L1 and FEZ-1 of the subclass B3 have the novel loop between α3-helix and β7-strand, and this flexible loop is located close to the active site (Figure 12c) [156,157].

**Figure 13:**
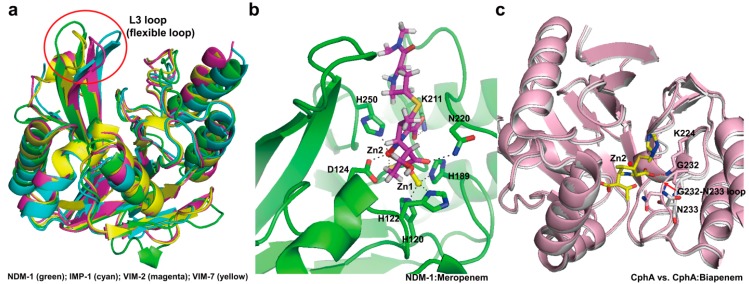
(**a**) Comparison of the structural features of NDM-1 (PDB entry 3SPU, green) with IMP-1 (PDB entry 1DD6, cyan), VIM-2 (PDB entry 1KO2, magenta), and VIM-7 (PDB entry 2Y87, yellow), showing the significant differences in orientation of the L3 loop. L3 loops are indicated by the red circle; (**b**) Active site of the NDM-1 in complex with meropenem (PDB entry 4EYL, green) is shown. The residues (H120, H122, D124, H189, K211, N220, and H250; NDM-1 numbering) in the active-site cleft are shown as green sticks. Zinc coordination and hydrogen bonding in active site in NDM-1 are shown as dashed black lines. The hydrolyzed meropenem is shown in magenta. Zn1 and Zn2 are represented as yellow and red spheres, respectively; (**c**) The conformational change upon substrate binding is represented by superimposition of the wild-type CphA (PDB entry 1X8G, white) and CphA (N220G mutant):biapenem complex (PDB entry 1X8I, light pink). The residues (K224, G232, and N233; class B numbering) in the active-site cleft are shown as sticks. The movement (from open to closed position) of G232-N233 loop appears as a red arrow. The closed position is shown in CphA (N220G mutant):biapenem complex structure. The hydrogen bond interactions between the C3 carboxyl group of biapenem and two residues (K224 and N233) in the active site in CphA are shown as dashed red lines. The hydrolyzed biapenem and Zn2 are represented as yellow stick mode and red sphere, respectively. Superpositions were performed using SSM Superpose [53] to align the complete chains. The structures of NDM-1 and CphA were prepared using *PyMOL* [54] and data adapted from King *et al.* [149] and Garau *et al.* [154], respectively.

NDM-1 shows lower sequence identity with other MBLs, and the most closely related MBLs are VIM-type and IMP-type enzymes, which show 37% sequence identity with NDM-1 [150]. Although the overall structure of NDM-1 has a common feature that exists in other MBLs structures, there are some differences. The L3 loop in NDM-1 was revealed to be more open and hydrophobic than that in IMP-1, VIM-2, and VIM-7 (Figure 13a). It suggested that the loop L3 may play an important role in the binding of the antibiotics at the active site [150] as previously described in BcII (Figure 13a). The crystal structure of NDM-1 bound to meropenem has been determined recently [149]. The hydrolyzed carbapenem core of meropenem had extensive non-covalent interactions with the zinc center and the R1 side chain of meropenem is positioned on the active-site cleft (Figure 13b). Although the pyrrolidine *N*,*N*-dimethylcarboxamide (DMP) R2 side chain of meropenem did not interact with NDM-1, the large NDM-1 active-site cleft provided steric accommodation of the bulky R2 side chain of meropenem, leading to the recognition and hydrolysis of meropenem. It has been previously suggested that the highly conserved Asn233 (class B numbering) of subclass B1 MBLs has a role in forming an oxyanion hole to facilitate the hydrolysis and substrate binding and stabilization of the tetrahedral intermediate via interaction between δNH_2_ of Asn220 and the β-lactam carbonyl oxygen of substrates [153,169]. In the structure of NDM-1 in complex with meropenem, the δNH_2_ of Asn233 (Asn220 in NDM-1) made the hydrogen bond with the C6 carboxylate oxygen of meropenem (Figure 13b). In addition, mutagenesis and kinetic analysis of IMP-1 enzyme have revealed that the Asn233Ala and Asn233Glu mutants appeared significantly increased *K*_m_ and reduced *K*_cat_ values for imipenem compared with the wild-type [174]. Taken together, it suggested that Asn233 in MBLs may play an important role in carbapenem binding and hydrolysis [149]. In subclass B2 enzymes, the crystal structure of CphA Asn220Gly (class B numbering) mutant in complex with biapenem has been first determined. Superimposition of wild-type CphA and CphA (N220G mutant):biapenem complex structures revealed the conformational change (movement from open to close position) of the loop including Gly232 (class B numbering) and Asn233 residues located at the entrance of the active site (Figure 13c), implicating that the Gly232-Asn233 loop may exist in the open position before substrate binding. In addition, the C3 carboxyl group of biapenem formed strong hydrogen bonds with the side chain of Lys224 (Lys211 in NDM-1, Figure 13b) and the backbone nitrogen of Asn233. This result also suggested that Asn233 in CphA may also have an effect on carbapenem binding and hydrolysis.

## 4. Experimental Section

**Figure 14:**
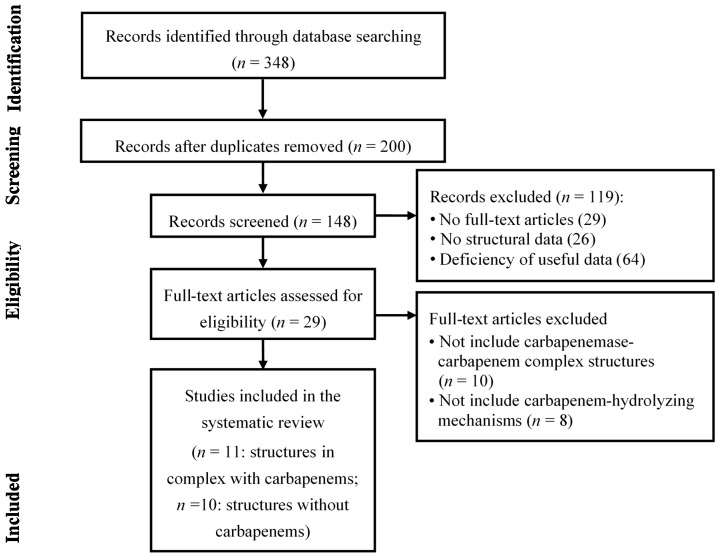
Literature selection process (PRISMA flow diagram). A total of 11 articles among 29 articles include structures in complex with carbapenems and mechanisms of carbapenemases. Ten articles among 29 articles include structures without carbapenems and mechanisms of carbapenemases. There were eight articles among 29 articles that include structures of carbapenemases without mechanisms. A total of 21 articles were included in the systematic review.

We used the Preferred Reporting Items for Systematic Review and Meta-Analysis (PRISMA) in our review (Figure 14) [175]. We conducted a systematic literature search in the following databases PubMed, Medline, and Embase. We used keywords as search terms. We combined terms for selected indications (structure and carbapenemase or metallo-β-lactamase). The literature search included all studies published in English between 2000 and 2014. We identified 348 references after removing duplicates. We independently assessed full-text articles for inclusion in our review. To find articles showing carbapenemase-carbapenem complex structures, the criteria for inclusion of studies encompassed crystal structures of the complexes and carbapenem-hydrolyzing mechanisms of the four classes of carbapenemases. After discarding 18 articles, we identified three articles involving class A carbapenemase, two articles involving class B carbapenemase, one article involving class C carbapenemase, and five articles involving class D carbapenemase. We found 10 articles showing carbapenemase structures and carbapenem-hydrolyzing mechanisms without carbapenemase-carbapenem complex structures (Figure 14).

## 5. Conclusions

This systematic review provides the structural basis to explain carbapenem-hydrolyzing mechanism at the atomic level. Unlike the serine dependent β-lactamases (classes A, C, and D), all class B β-lactamases are carbapenemases that can catalyze the hydrolysis of carbapenems. The main structural features of MBLs compared with serine-dependent carbapenemases are the presence of a zinc ion, the absence of covalent acyl-enzyme intermediate, and the widen active-site cleft. These characteristics could result in easier access of the long R2 side chain of carbapenem, more effective coordination of the water molecule, and fast breakdown of the tetrahedral intermediate through protonation of the nitrogen of the cleaved β-lactam ring, which may be the reason that all MBLs can hydrolyze carbapenems.

From the analysis of recent three-dimensional structures of carbapenemases in complex with carbapenems, the two main mechanisms by which serine β-lactamases acquire the carbapenemase activity are identified as follows: first, increased activity of the water molecule through (i) the change of position of the water molecule; (ii) a loss of the hydrophobic interaction; or (iii) a loss of the hydrogen bond between a water molecule and the R1 side chain of carbapenem; second, increased access and stabilization of the R1 and R2 side chains of carbapenem through (i) the positional shift of active-site residues; (ii) the interaction between residues in the active site and the R1 and R2 side chains; (iii) the structural change of the important loops (e.g., C-, R2-, β5–β6, L3 loops) surrounding the active site; (iv) structural rotation of the carbapenem-packing conformation; or (v) the formation of hydrophobic tunnel-like pore interacting with the R1 and R2 side chains. Additionally, it has been suggested that the difference of tautomerization of the pyrroline ring of the cleaved β-lactam ring is involved in acquiring the carbapenemase activity through most of the known various mechanisms such as integron and transposon. The class D β-lactamases have been most extensively studied, and acquired carbapenemase activity through most of the known various mechanisms. On the other hand, in the class C β-lactamases, the only carbapenemase activity-acquiring mechanism studied in CMY-10 and ADC-68 is the structural change of the C- and R2-loops that increases the access of the R2 side chain of carbapenem. Notably, class C β-lactamases acquire carbapenemase activity through the change of residues involved in the R2 side chain of carbapenem, whereas the carbapenemase activity-acquiring mechanisms of class A and D are mainly related to the conformational change of the R1 subsite which accommodates the R1 side chain of carbapenem.

The major structural feature of class A carbapenemases is that their structures did not show a large structural difference compared with non-carbapenemases, except for the disulfide bridge between Cys69 and Cys238. However, it was observed that the disulfide bridge induced the conformational movements of specific residues (Ser70, Ser130, Asn132, and Asn170) in the active site. These alterations in the active site could permit accommodation of the 6α-1*R*-hydroxyethyl group of carbapenems. In addition, in SME-1 and GES-5, it has been proposed that the conformational change of the important residue surrounding the active site could induce an increased activity of the water molecule by the positional change of the water molecule or the formation of new hydrogen bond with the water molecule. Another proposed mechanism was also involved in the availability of the water molecule. The 6α-1*R*-hydroxyethyl group in SFC-1 and GES-5 interacted with Asn132, while the 6α-1*R*-hydroxyethyl group in non-carbapenemases interacted with the deacylating water molecule. Because it was proposed that the interaction of the deacylating water with the 6α-1*R*-hydroxyethyl group induces inactivation of the water molecule, this configuration in SFC-1 and GES-5 may lead to efficiently hydrolyzing carbapenem.

In class C carbapenemase, the mechanism involved in the structural change of the C- and R2-loops was only proposed. A three-amino-acid deletion in the R2-loop of CMY-10 and ADC-68 significantly enlarged the R2 subsite. The ADC-68 C-loop was stabilized in the open conformation for the upper R2 subsite, by which carbapenems with bigger R2 side chains could be accommodated. Accordingly, the modification of the R2 subsite in class C carbapenemase may enhance catalytic efficiency against these clinically important β-lactams.

Class D carbapenemases exhibit the most various structural changes compared with non-carbapenemase. OXA-23, OXA-24/40, OXA-48, and OXA-58 are representatives of class D carbapenemases. In OXA-24/40, a hydrophobic bridge formed by Try112 and Met223 led to the stabilization of the Δ^2^ tautomeric form of doripenem, which may play a pivotal role in carbapenemase activity; however, OXA-48 and OXA-58 carbapenemases had no hydrophobic bridge in the active site and showed a differently shaped active site. Moreover, although OXA-23 had the hydrophobic bridge formed by Phe110 and Met221, the meropenem in OXA-23 was present as the Δ^1^ tautomeric form and the hydrophobic bridge has less impact on the carbapenemase activity. Thus, the hydrophobic bridge does not seem to be the sole mechanism for carbapenem hydrolysis by class D carbapenemase. In addition, in OXA-23, it has been proposed that a loss of the hydrophobic interaction between Val128 and Leu166 may create a space to access hydrolytic water into the active site, which may lead to increased activity of the water molecule. In OXA-23, OXA-24/40, OXA-48, and OXA-58, a different mechanism was suggested. The formation of narrow active-site cleft by the shortened β5–β6 loop played an important role in determining carbapenemase activity, and mutagenesis studies have confirmed the role of the β5–β6 loop in carbapenemases. However, the exact reason why the formation of narrow active-site cleft poses carbapenemase activity was not determined. Interestingly, the catalytic efficiencies of OXA-48 and OXA-58 for imipenem are higher than those of OXA-23 and OXA-24/40. Therefore, these structural studies revealed that these class D carbapenemase subgroups may each have a different mechanism in acquiring carbapenemase activity. OXA-146 is an OXA-23 subgroup member with the insertion of a single alanine residue (A220) in the β5–β6 loop. Despite the single amino acid difference, OXA-146 was able to hydrolyze extended-spectrum cephalosporins with a good efficiency comparable with those of other extended-spectrum β-lactamases. The overall structures of OXA-146 and OXA-23 showed that the structural alteration caused by a single insertion in the β5–β6 loop, the movement of the Met221 residue out of its normal position, is likely to relieve steric clashes between the bulky R1 side chain of ceftazidime and Met221 in OXA-23.

In class B β-lactamases, crystal structures of various carbapenemases such as NDM-1, CphA, and IMP-1, were identified. In NDM-1 and CphA, the hydrolyzed core of carbapenem displayed extensive non-covalent interactions with the zinc center, but the R2 side chain of carbapenem did not exhibit any strong interaction. These results suggested a reason why all class B β-lactamases are carbapenemases. At the least, the easy access of the long R2 side chain of carbapenem and the extensive non-covalent interactions of the β-lactam ring with the zinc center could be one of the important reasons of this phenomenon.

Based on carbapenem-hydrolyzing mechanisms obtained from the crystal structures of the four classes of carbapenemases, novel drugs for the treatment of a carbapenemase-producing pathogen have to be developed considering the following points: (i) the carbapenem-hydrolyzing mechanisms of class A and D carbapenemases are mainly related to the conformational change of the active site which accommodates the R1 side chain of carbapenem; (ii) the carbapenem-hydrolyzing activity in class C carbapenemase is caused by the widened R2 subsite which accommodates the R2 side chain of carbapenem; and (iii) because the R2 side chain of carbapenem did not exhibit any strong interaction with class B carbapenemases, the design of the novel mechanism-based inhibitors (or MBL-resistant β-lactam antibiotics) should consider the R1 side chain of carbapenem. The expansion of our knowledge of enzyme structure and insight into the action mechanism of carbapenemases may assist the design and development of new drugs for minimizing the spread of antibiotic resistance.
